# Supplementary material for: Patterns of Intron Gain and Loss in Fungi
Source: PLoS Biol. 2004 Nov 30;2(12):e422. doi: 10.1371/journal.pbio.0020422 (PMC532390; doi:10.1371/journal.pbio.0020422)
Supplement: Table S1 — Also available at http://genes.mit.edu/NielsenEtAl/. (4.3 MB ZIP). [file pbio.0020422.st001.zip › NielsenEtAl/html/474.html]

AN4239.1.NCU07290.1.MG05378.1.FG01193.1


```
 CLUSTAL W (1.82) Multiple Sequence Alignments - Introns Inserted


Sequence 1: NCU07290.1	527 aa
Sequence 2: MG05378.1	536 aa
Sequence 3: FG01193.1	520 aa
Sequence 4: AN4239.1	595 aa
Alignment Length: 616 aa
Number Identitical Residues: 198 aa
Alignment Score (without introns) 10497


MG05378.1 	MDEDEPMADAAPEAEPSKD~NE-------DEEEDPAHEPE--------------AEDDDD
NCU07290.1	MPSRRSGRAAAKRAQQALE1STPKTFEGLDDEDEPMPDAD--------------QDDAEP
FG01193.1 	MAPRGKIRLRLTRSAKS-E~STPKSFADLEDEPMPDASPS--------------AHASRR
AN4239.1  	MP--RKLRAAAQAAAQSMK1NVAPPLGDGSDEEMIEAPPSRESSAPVVPDEAEDEEDAKE
          	*            :  : . . . .  . .:*      .. .:::.  ...:.. .    

MG05378.1 	ENKSGGDEDEDD-----EQP-----AEEAEDDEEEAAKSPS----PPPVQ----------
NCU07290.1	SQRDGEDDVEKD-----DESGGDGHEEEEAPEEDESGKSPS----PPPEP----------
FG01193.1 	HQVPDPDDEKDEDHQPKDESGDEDDAADAAEKDDESVKEPT----PPPQP----------
AN4239.1  	GENAGKEEQEGASSSKAEEPDTPAQPALAQGEGEEAAATPAQDSNPPSRPDTPTHLEAGR
          	 :  . :: :  . .  ::..          . :*:   *:..:.**.  .:.:  .:. 

MG05378.1 	---TVRRKRLGRPPKNRPPDWDNM-IEVPVREGEQNTPRRRGR-GGWRGRGGRKGASLAP
NCU07290.1	---VIRRRRLGRPPKNRPPDWDTLPIEPPNRD--ENAPRRRGR-GGWRGRGGRKGQHFQP
FG01193.1 	---MVRRKRLGRPPKNRPPDWDNM-ISVPADE--ANTPRRRGR-GGWRGRGGRKGGPAAP
AN4239.1  	VSAIPRKRRIGRPPKNRPPDWDAPADGSPQIHVSTPVKRRRGRPAASGGRWGRGRGPSHV
          	 ::  *::*:************  .   *  . .  . *****...  ** **       

MG05378.1 	TQQAIDKEGTMMDIINDEVALPEDPEGETKVDKNGNLQGGREYRCRTFTVAGRGDRLYML
NCU07290.1	TQQSIDKDGTVLDIINDEVDLPEDPEGEKKVDKLGNLQDGREYRCRTFTVLGRGDRLYML
FG01193.1 	KAEQVIDDGSIVEIVNDECVLPEDPEGETKVDKSGNLQGGRDYRCRTFTVSGRGDRLYML
AN4239.1  	TQVPIDKEGNMMDVIDDEVAVPGDPEGDTKVDKNGILQGGREYRVRTFTILNRGERQYML
          	.   : .:*.::::::**  :* ****:.**** * **.**:** ****: .**:* ***

MG05378.1 	STEPARCVGFRDSYLFFTKHLRLTKVIVDDEEKRDMIEREIIPHSYKGRSIGIVTARSVF
NCU07290.1	STEPARCVGFRDSYLFFTKHRKLYKIIVNDEEKRDMIEREIIPHSYKGRTIGIVTARSVF
FG01193.1 	STEPARCVGFRDSYLFFTKHKKLYKIIVDDDEKRDMIERDIIPHSYKGRSIGIVTARSVF
AN4239.1  	STEPARCIGFRDSYLFFQKHKLLYKIIIDDDAKRDLIERDIIPHSYKGRAIGVVTARSVF
          	*******:********* **  * *:*::*: ***:***:*********:**:*******

MG05378.1 	REFGALIIVGGRRIIDDYEVGKARMDGVVEGELADPNDHFVV-GEPYNKNQYVAWHGASS
NCU07290.1	REFGALIIVGGRRIIDDYEVAKAREEGVVEGELADPNDIFEP-GKPYNKNQYVAWHGASS
FG01193.1 	REFGARIIVGGKRILDDYNVALARAEGVIEGEIADPNDRFIE-GEPYNKNQYVAWHGASA
AN4239.1  	REFGAKIIVGGRKVIDDYQAQAARERGDVEGELAVPEDKLPPPGEPYNKNQYVAWHGASS
          	***** *****::::***:.  **  * :***:* *:* :  .*:**************:

MG05378.1 	VYHSAAPVVAQQTGVFVAP-KKRRVAVNDTNWMYEHAKEAS2NFNSILTGMRQANLHGVY
NCU07290.1	VYHSNIPSVPQQN-AKVAP-TKRRVAVNDVNWQLEHAREAS2NFNSILTSVRRANLNGVY
FG01193.1 	VYHTNVPSVPVPN-GKIES-RKRKVNVNDMNWMLEHAREAS2SFNASINAIRKLNNAGVY
AN4239.1  	VYHTSTPAVPIPGTGKVVDSKKRRVTVTGDNWMLEHAREAA2NFNAVLSHTRQQNLGGVY
          	***:  * *.      :  : **:* *.. **  ***:**: .**: :.  *: *  ***

MG05378.1 	DMHTNMMHYPATMQPTHARIEVVPLEQQQ--------------------------QHEQP
NCU07290.1	DIHTNQMHYPHIIQPTHARITQVVDSE---------------------------------
FG01193.1 	DIHTNVMQYPVTMQPTRARIEQVAPEDE--------------------------------
AN4239.1  	DIHTNIIHYPKIMQPTHARWERVPPSDARGANKLTKEMSTLTLSNGVVEQENAPPEPETE
          	*:*** ::**  :***:**   *  .:  .:.. :.. :: : :..  ....:... .  

MG05378.1 	REQDNDSASTSSIFKPLPSTVPRNFRVVDTVLETPPSGIAPSSYDRRFFID-GDRAGGGG
NCU07290.1	-----DASSSSSDLPLVKPSISRNFLITDVEIELPPAGISPAAYEVPFRTSPADRA----
FG01193.1 	-----EATADSSVFRPVPAKMARNFFVADTYFETSSAGVISASGVDGTVSS---------
AN4239.1  	IQDSKPAGETSTIFSQVPSALARRFAVLDFYTESPTYSNLGIPGPDGDVHDLGSNG----
          	 .... :   *: :  : . :.*.* : *   * .. .    .       . .. .    

MG05378.1 	SSSTRDRGSTDFLAPFRGLSAVSDEIRDLLPAECRAAFDQAQQRERDWHSKWGPEKDAAC
NCU07290.1	-ASAR----ADFLAPFKGLSAVPDDIRDLLPEECRKAFDSAVKNEEEWFGKWGDEKDTTC
FG01193.1 	---------ADFLASFQGLSTVSDDIKDLLPPECRAAFDNAVEKETSYRSQWGPETENMS
AN4239.1  	---------LISVANPKHPEFVNPEILDELPPDCKEALIEAASHEWEWKSRWCSEVDDGA
          	            :*  :  . *  :* * ** :*: *: .* ..* .: .:*  * :  .

MG05378.1 	RRDPVIDKAIVPMVKMV-
NCU07290.1	RREPVIDKAIVPYSMNIM
FG01193.1 	RRQPIIDKAIVPYSMS--
AN4239.1  	RVAPRKSYAWFP------
          	*  *  . * .*
```
